# Does the political will exist to bring quality-assured and affordable drugs to low- and middle-income countries?

**DOI:** 10.1080/16549716.2019.1586317

**Published:** 2019-04-15

**Authors:** Eduard J. Beck, Sundhiya Mandalia, Boniface DongmoNguimfack, Eloan Pinheiro, Ellen ‘t Hoen, Pascale Boulet, John Stover, Aashta Gupta, Sandeep Juneja, Vincent Habiyambere, Peter Ghys, Cesar Nunez

**Affiliations:** a Programme Branch, UNAIDS, Geneva, Switzerland; b NPMS-HHC CIC, London, UK; c WHO, Geneva, Switzerland; d Pinheiro Consulting, Rio de Jainero, Brasil; e Medicines Law & Policy, Geneva, Switzerland; f Avenir Health, Washington, DC, USA; g Medicines Patent Pool, Geneva, Switzerland

**Keywords:** Trade-related aspects of intellectual property rights (TRIPS), intellectual property rights and patents, annual originator and generic HIV drug cost estimates, low-and middle-income countries

## Abstract

**Background**: Increased coverage with antiretroviral therapy for people living with HIV in low- and middle-income countries has increased their life expectancy associated with non-HIV comorbidities and the need for quality-assured and affordable non-communicable diseases drugs . Funders are leaving many middle-income countries that will have to pay and provide quality-assured and affordable HIV and non-HIV drugs, including for non-communicable diseases.

**Objective**: To estimate costs for originator and generic antiretroviral therapy as the number of people living with HIV are projected to increase between 2016 and 2026, and discuss country, regional and global factors associated with increased access to generic drugs.

**Methods**: Based on estimates of annual demand and prices, annual cost estimates were produced for generic and originator antiretroviral drug prices in low- and middle-income countries and projected for 2016–2026.

**Results**: Drug costs varied between US$1.5 billion and US$4.8 billion for generic drugs and US$ 8.2 billion and US$16.5 billion for originator drugs between 2016 and 2026.

**Discussion**: The global HIV response increased access to affordable generic drugs in low- and middle-income countries. Cheaper active pharmaceutical ingredients and market competition were responsible for reduced drug costs. The development and implementation of regulatory changes at country, regional and global levels, covering intellectual property rights and public health, and flexibilities in patent laws enabled prices to be reduced. These changes have not yet been applied in many low- and middle-income countries for HIV, nor for other infectious and non-communicable diseases, that lack the profile and political attention of HIV. Licensing backed up with Trade-Related Aspects of Intellectual Property Rights safeguards should become the norm to provide quality-assured and affordable drugs within competitive generic markets.

**Conclusion**: Does the political will exist among policymakers and other stakeholders to develop and implement these country, regional and global frameworks for non-HIV drugs as they did for antiretroviral drugs?

## Background

The UNAIDS *Fast Track Initiative* includes the *90–90–90 targets* [1,2] to ensure that most people living with HIV (PLHIV) by 2030 have been diagnosed, are in care and receive antiretroviral therapy (ART) with undetectable viral load. ART reduces HIV-related morbidity, increases the life expectancy of PLHIV [3] and decreases HIV transmission, reducing the number of people newly infected with HIV [4]. If PLHIV start ART at the time of diagnosis, the number of PLHIV on ART is expected to increase in those countries that have implemented the 2015 World Health Organization (WHO)  guidelines [5]. Increased life expectancy will increase the number of older PLHIV on life-long ART, many of whom are likely to develop non-communicable diseases (NCDs) [6,7].

Costs for first-line ART decreased over the last two decades from US$10,000–15,000 per annum for originator-based triple therapy to US$64–102 per annum for generic ART [8] as a consequence of the increased use of generic antiretroviral drugs (ARVs) and market competition. Until recently, many low- and middle-income countries (LMICs) had the supply and cost of ART covered by donor agencies. The President’s Emergency Program for AIDS Relief (PEPFAR) and the Global Fund for AIDS, Tuberculosis and Malaria (GFATM) are now transiting out of many LMICs and focusing on HIV high-burden countries [9]. Many middle-income countries will increasingly have to rely on domestic sources to pay for ART. International funding for HIV fell from US$7.5 billion in 2015 to US$7.0 billion in 2016, a decline of $511 million (7%), bringing disbursements to their lowest level since 2010 [10]. The current US administration requested US$4.3 billion for bilateral HIV funding in 2018, which is down from US$5.2 billion in 2017 [11].

**Figure 1.**
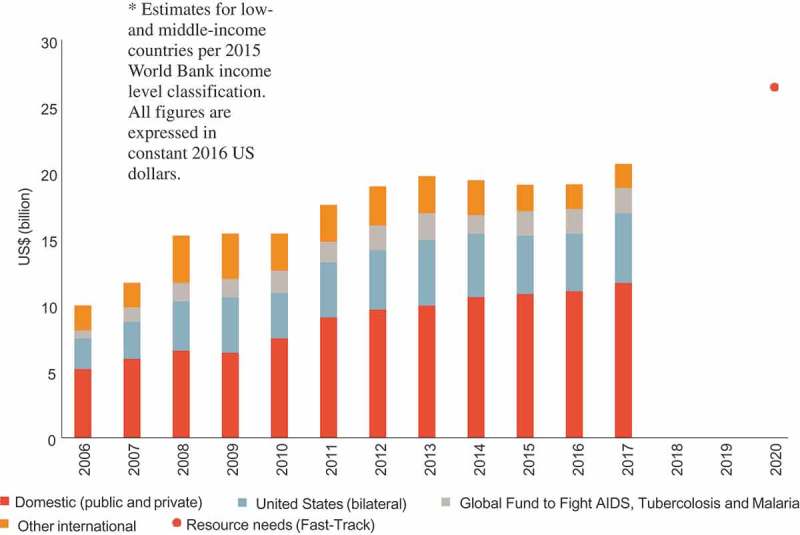
Annual financial resources available to low- and middle-income countries in 2016 US dollars for their HIV response from internal and external sources of funding, 2006−2017, and the estimated resources needed (resource need) for a successful implementation of the Fast Track Strategy by 2020 [13].

Various mechanisms have been employed to speed up access to quality-assured, affordable generic ARVs. Such mechanisms include the use of Trade-Related Aspects of Intellectual Property Rights (TRIPS) flexibilities and license agreements negotiated through the Medicine Patent Pool (MPP) [12]. Both licensing mechanisms focus on maximizing generic competition, economies of scale gained through high-volume procurement, and other efficient procurement and distribution strategies. Some, but not, all LMICs have increased their domestic expenditure on HIV. As ART coverage continues to expand, it is uncertain whether many countries will be able to compensate for the reduced donor funding and thus may not be able to reach Fast Track financial commitments by 2020 (Figure 1).

This paper provides estimates of originator and generic ARV costs in LMICs for the years 2016–2026. The discussion describes the country, regional and global mechanisms used to reduce the price of ARVs and identifies which mechanisms are required to sustain the production and supply of quality-assured and affordable ARVs, drugs for other communicable diseases and NCDs in LMICs.

## Methods

Projections of the number of people receiving ART by regimen and line of therapy in 2016–2016 were based on analyses by the WHO, UNAIDS and partners [14–16]. To project future demand for ARVs in LMICs through to 2026, a consolidated forecast was used based on data of past coverage and current demand from multiple sources. These included: (1) WHO, the Antiretroviral Medicine and Diagnostic Use Survey [17,18], (2) projections from the Clinton Health Access Initiative (CHAI), (3) the Global Price Reporting Mechanism of WHO (GPRM), and (4) the Global AIDS Monitoring (GAM) system for HIV testing and linear extrapolations of past coverage. Data from 130 countries were analyzed, including 114 LMICs and 16 high-income countries (HICs). For countries not reporting in this survey, coverage was estimated. Historical data on the number of people receiving ART were obtained from the UNAIDS AIDS info database [19]. Annual growth in ART patient numbers were based on the Fast Track projection [20]. A technical working group composed of WHO, the United Nations Children’s Fund (UNICEF), UNAIDS, the USA Centers for Disease Control and Prevention (CDC), the US Agency for International Development (USAID), CHAI, and Avenir Health reviewed the results and provided feedback about data quality, assumptions for projection methodology, and review of the final results.

Procurement data and prices of originator and generic drugs held in the GPRM [8] were combined with projections of the number of PLHIV on first, second and third lines for different ART combinations [14–16]. Weighted annual prices for each of the individual ART combinations were estimated for the number of the people on a particular ART combination at each year from 2016 to 2026. Annual prices for all combinations were added to produce originator and generic costs per annum for different lines of therapy. For the study period, annual costs were added to provide total annual costs based on originator and generic prices across all lines of ART. No assumptions were made regarding potential future reductions in the cost of ARVs due to increased volume. Total weighted population costs of both the originator and the generic ART costs are presented as annual US dollars (US$) for the years 2016–2026.

## Results

**Figure 2.**
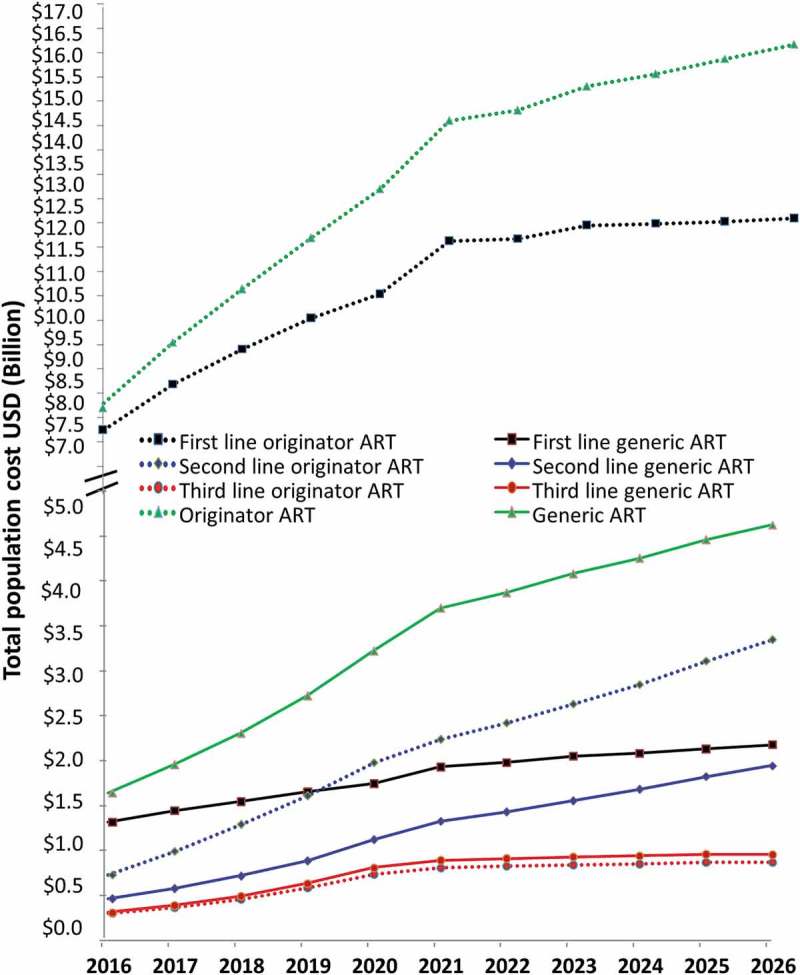
Projections 2016–2026 of originator and generic prices comparing population costs in US dollars (US$) for people on first-, second-, and third-line antiretroviral therapy (ART) and across all lines of therapy (broken axis).

Costs for ART were projected to increase from US$8.2 billion to US$16.5 billion per annum for originator drugs and from US$1.5 billion to US$4.7 billion for generic drugs between 2016 and 2026 (Figure 2). Originator drugs resulted in higher costs compared with generic drugs for both first- and second-line ART. For third-line therapy there was not much different in cost; in fact, the generic versions were slightly more expensive than the originator drugs (Figure 2).

In 2017 the number of PLHIV on ART was 21 million [21]; therefore, these estimates are likely to underestimate the annual resource needs if ART coverage continues to increase at the same rate and prices remain as they are. As the number of PLHIV on ART increases, so will the annual cost for ART in LMICs unless price reductions continue to be achieved in the future.

## Discussion

The necessary country, regional and global mechanisms required to ensure quality-assured and affordable drugs include improving the cost-effectiveness of producing and distributing ARVs, and developing and implementing the necessary regulatory frameworks to ensure the existence of competative markets for originator and generic drugs.

### Country mechanisms

#### Factors determining ARV prices

Active pharmaceutical ingredients (API) costs contribute 60–80% of the overall cost of ARVs [22,23]. The remainder of the costs are due to the production of the final formulation and a profit margin (Table 1). The production costs for APIs are related to the cost of energy, solvents, labor and capital investment, and raw materials. To minimize operating cost, economies of scale need to be maximized that are influenced by the availability of raw materials. The major suppliers of raw materials are Chinese companies, many of which do not request Good Manufacturing Practice (GMP) compliance [24]. The production of APIs is dominated by Indian companies. Ideally, the synthesis of APIs uses inexpensive raw materials that can be employed for multiple purposes and are easily available. When APIs require uncommon raw materials, this increases the cost of manufacturing [22]. The efficiency of production processes is also important: ‘Current production processes are more efficient…the cost of EFV [Efavirenz] API production has fallen from as high as USD425/kg to about USD17–20/kg today’ [22].

**Table 1.** The categories comprising the direct and indirect production costs and operating margin for drug manufacturing.

Direct manufacturing cost	Indirect cost manufacturing cost overhead	Indirect cost non-operational overhead	Operating margin
Active pharmaceutical ingredients (API)	Indirect materials	Administration	Funds for sustainability
Equipment	Indirect labor	Research and development	Funds for future growth and development
Labor	General utilities	Human ﻿Resources	
Excipients	Insurance	Marketing	
Packing			
Loss in process			

Given the competitive nature of the market, API manufacturers usually do not publicize their production costs as economies of scale are important. However, overall, APIs for ARVs are sold at roughly 20–40% above their production cost [25]; as the availability increases, prices decrease (Table 2). The nature of the production plant also affects the price of APIs. Some can keep their prices down because of large production volumes, while others are both pharma-chemical and pharmaceutical companies producing both APIs and finished formulations. The latter produce their APIs in-house, which are therefore cheaper and have less impact on the price of the finished product compared with a manufacturer that had to buy the API [23].

**Table 2.** Antiretroviral (ARV) prices on the pharmaceutical market and estimates of the lowest price of active pharmaceutical ingredients (APIs), in US dollars [8,26,27].

Prices per year	Originator prices	Generic on the market	MSF prices	Estimates of the lower price of API on the market
**First line**				
NVP + AZT + 3TC	361	90	94	119
NVP + (TDF + 3TC)	482	64	124	88
NVP + (TDF + FTC)	519	79	88	107
EFV + (AZT + 3TC)	398	102	164	145
EFV + TDF + 3TC	519	80	106	97
EFV + TDF + FTC	613	77	100	108
DTG + TDF + 3TC	482	83	NA	204
**Second line**				
LPV/r + (AZT + 3TC)	392	282	316	399
LPV/r + (TDF + 3TC)	513	255	307	344
LPV/r + (TDF + FTC)	550	270	307	355
DTG + (LPV/r)	431	258	NA	434
ATV/r + (AZT + 3TC)	656	265	286	318
ATV/r + (TDF + 3TC)	777	238	277	263
ATV/r + (TDF + FTC)	814	253	277	274
DTG + (ATV/r)	695	241	NA	353
DRV/r + (AZT + 3TC)	892	1.074	NA	823
DTG + (DRV/r)	931	1.050	NA	873
**Third line**				
Ral + DRVr	1.046	1.615	NA	1696
DTG + DRVr	931	1.050	NA	873
Raltegravir	675	608	973	970

NVP= Nevirapine; AZT= Zidovudine; 3TC= Lamivudine; TDF = Tenofovir; FTC= Emtricitabine; EFV= Efavirenz; DTG = Dolutegravir; LPV/r = Lopinavir/ Ritonavir; ATV/r = Atazanavir/ Ritonavir; DRV/r = Darunavir/ Ritonavir; Ral+DRVr = Raltegravir + Darunavir; MSF = Medicin Sans Frontieres; NA = not available.

#### Cost structure for HIV drugs

Prices of certain APIs have decreased over the last decade, due to the increase in the production of ARVs and increased use of generic ART, especially in LMICs. However, ‘Price reductions for first-line ART APIs cannot continue indefinitely… many of these APIs are reaching a point of diminishing returns for continued cost reduction’ [22].

Using *Standard Cost Methodology* [24], a price for the final product can be determined, since the costs of the main steps of the productive process are known. The factory price of any drug is the sum of the direct manufacturing costs to which is added the indirect cost and operating margin (Table 1). Some pharmaceutical companies claim that the high prices of drugs are due to considerable investments in advertisement, marketing and taxes. Therefore, as long as public laboratories are exempt of such expenses, significant price reductions could be offered. The experience of Farmanguinhos has shown that a major reason for elevated prices for HIV drugs was the cost of the API [28].

Pharmaceutical companies invest in the development and testing of their drugs including by funding clinical trials; however, the extent to which costs are borne by industry or public subsidies varies by product, company, country where the drug is being developed, the strength of the scientific community enabling clinical trials to be conducted, and available resources. It is recognized that the development and production of many drugs has involved significant public support and resources [29]. Furthermore, pharmaceutical companies also spend a large amount of money on advertising. For instance, in 2016 US$6.7 billion was spent on direct-to-consumer pharmaceutical advertising alone in the USA [30]. Furthermore, ‘the number of drug brands with marketing budgets of $50 million or more in yearly spending nearly doubled between 2012 and 2015…and the number of budgets with $75 million or more rose from 13 to 30 during that time period’ [30].

#### Generic drugs as ‘specialty drugs’

Generally, generic drugs are priced lower than originator drugs, though some third-line generic ARTs in LMICs are currently comparable in price to or more expensive than originator drugs (Figure 2). This is related to the fact that the volume of drug required is currently not high; however, with increased demand and increased competition over time the price ought to come down.

The prices of generic off-patent drugs can be manipulated in other ways. Established off-patent generic drugs can be bought by companies and turned into high-priced 'specialty drugs’ when they have exclusivity in the market. Price increases are aided by a controlled distribution strategy where the supply of drugs is sold directly to consumers rather than through a network of retailers. This is a recognized marketing strategy that is employed by some companies to thwart the production and supply of other generics [31].

The drug Daraprim® or pyrimethamine was approved by the Federal Drug Administration (FDA) in 1953. Its marketing rights in the US were held by GlaxoSmithKline (GSK) until 2010, when they were sold to CorePharma. In 2011 annual income from sales of the drug jumped from US$667,000 to US$6.3 million, while the number of prescriptions remained around 12,700 [32]. Annual income rose to US$9.9 million in 2014, despite a reduction in the number of prescriptions to 8821, excluding in-hospital use of the drug. In 2015, Daraprim was sold to Turing for US$55 million, after Impax Laboratories had bought CorePharma in 2014. The price of a 25-mg tablet of Daraprim was then raised by Turing from US$13.50 to $750 per tablet [31]. Daraprim®, however, is not the only example of price increases associated with older generic drugs [33].

The drug benznidazole (BZN) was developed by Roche for the treatment of Chagas disease. In 2003 the production rights were transferred to the Brazilian government and BZN was produced by the Pernambuco State Laboratory (LAFEPE). To ensure ongoing supplies of API, the private company NORTEC was engaged by LAFEPE to produce the API, but LAFEPE did not have a GMP certificate and therefore could not access markets outside Brazil. In 2011 the Argentinian company ELEA produced BZN based on APIs from the Argentinian company MAPRIMED. Due to a lack of competition, ELEA could expand its market and charged higher prices than LAFEPE: ELEA’s market price in 2015 was US$85.52 compared with LAFEPE’s US$38.32 and profits were well above the 14% margin quoted by the companies [34]. The bidding process that can now be provided through PAHO’s Strategic Fund is one mechanism that can increase competition and hopefully reduce prices [35], as ‘there is a need for competition in order to influence market regulation for the prices associated with BZN sales’ [34].

In 2018, 90% of drugs prescribed in the US were generics [36], yet drug costs continue to rise. US spending on prescription medicines in 2016 increased by 5.8% over 2015 levels to $450 billion based on list prices, and by 4.8% or US$323 billion when adjusted for discounts and rebates. Annual spending has been projected to increase by 4–7%, reaching US$580–610 billion in 2021 [37].

A study on prescription claims of 1120 generic drugs from US commercial health plans, 2008–2013, reported that drugs produced by four generic companies had a 31.7% reduction in price, compared with an 11.8% reduction if produced by two companies during the study period [38]. Conversely, drugs that were produced in near-monopoly or monopoly levels of competition increased by 20.1% and 47.4% in price, respectively, during the study period (Table 3). The number of manufacturers of the generic drug was strongly associated with relative price [39]. Generic drugs with three or fewer manufacturers are at risk of entering into a shortage, and applications for drug products where this situation applies should be prioritized [40]. To develop a stable generic drug marketplace, the FDA implemented its Drug Competition Action Plan in 2017 [41]. It recently announced additional steps to encourage generic competition [42] and reported that it had approved its first generic drug under the new pathway aimed at enhancing market competition for sole-source drugs [43].

**Table 3.** Prescription claims generic drugs prices, US commercial health plans 2008–2013 [38].

Monopoly generic market	+ 47.4% (CI, 25.4% to 73.2%)
Near-monopoly generic market	+ 20.1% (CI, 5.5% to 36.6%)
Two generic companies	−11.8% (CI, −18.6% to −4.4%)
Four generic companies	−31.7% (95% CI, −34.4% to −28.9%)

+ increase in price; – decrease in price; CI = confidence interval.

Generic drug policies also have become the backbone of many other HICs' cost containment strategies for drugs with regulatory provisions to ensure entry into the market of generics as soon as patent and market exclusivities expire. Some countries also have incentives to ensure generic prescriptions and substitutions [44]. More recently there have been demands in HICs from both the medical profession and the public not to wait until the patent exclusivity has expired but to allow generic entry to ensure access using compulsory licensing or personal import by individual patients [45–47]. HICs as well as LMICs are now using the TRIPS regulatory flexibilities for a range of drugs [44,48], indicating the importance of developing and implementing country-based regulatory mechanisms.

### Global and regional mechanisms

The HIV pandemic presented the challenge of providing life-saving drugs for millions of PLHIV in LMICs in the late 1990s. High ARV prices were sustained by market monopolies through patents that gave the patent holder the right to exclude others from commercially exploiting the invention. The minimum patent term for medicines is 20 years, but market exclusivity can be extended beyond this period. Patents can be granted by national or regional government bodies [49]. The HIV pandemic focussed attention on the role of patents in maintaining the high prices of these new medicines. In 2000, ART was initially available in HIC countries while, at the same time, very few PLHIV in Africa had access to ART. ARVs were available only from originator companies that produced small quantities of expensive ARVs and charged prices between US$10,000–15,000 per person per year.

The exclusion of pharmaceutical product patents from the 1970 Indian Patents Act [50] allowed a generic industry in India to flourish, and in 2001 provided the legal framework for large-scale production of generic ARVs including fixed-dose combinations (FDCs) and paediatric formulations. The establishment of the early HIV treatment programs in Brazil and Thailand in the late 1990s and early 2000s was possible, in part, because key drugs were not patent protected and local producers could produce ARVs at much lower costs. These included zidovudine, lamivudine, nevirapine and stavudine. Brazil’s purchasing power assisted in reducing the world price of APIs that stimulated the large-scale, low-cost production of ARVs by Indian companies. This caused the price of APIs and ARVs to come down dramatically, as demonstrated by price reductions for ritonavir, lopinavir, darunavir, atazanavir, raltegravir and dolutegravir (Table 4).

**Table 4.** Variation of active pharmaceutical ingredient (API) prices to make antiretroviral (ARV) formulations, comparing the years 2005, 2010 and 2015 from various sources including the World Health Organization (WHO) [8,26].

API price for different ARVs of generic companies	WHO price in 2005 (US$)	WHO price in 2010 (US$)	Reduction of the price from 2005 to 2010 (%)	HO price in 2015 (US$)	Reduction of the price from 2010 to 2015 (%)	2015 generic producers' prices on the market (US$)	Reduction of the price (%)
Atazanavir	NA	1400	-	1000	29	921	8
Darunavir	NA	NA	-	1500	-	922	38
Efavirenz	1200	250	79	120	52	105	13
Emtricitabine	NA	320	0	280	13	231	18
Lamivudine	295	160	46	120	25	174	-
Tenofovir	3000	500	83	180	64	187	-
Ritonavir	2600	900	65	622	31	911	-
Lopinavir	2900	1150	60	408	65	526	-
Nevirapine	320	156	51	110	29	102	7
Zidovudine	360	260	36	230	12	186	19
Dolutegravir	NA	NA	-	5000	-	NA	-
Raltegravir	NA	NA	-	NA	-	3486*	-

NA = not applicable.

In many countries patent barriers continued to exist that prohibited the production and supply of generic ARVs. Patent-related trade and legal disputes broke out, including a legal challenge mounted by 39 drug companies in 1998 against the South African government over an amendment to its Medicines Act (see Box 1). Global public pressure resulted in this legal challenge being abandoned in 2001 and focused the world’s attention on pricing of drugs and intellectual property [52].

**Box 1.** Pharmaceutical Manufacturers’ Association of South Africa versus President of the Republic of South Africa. 1998 Case No 4183/98, filed 18 February 1998.

'In February 1998, the South African Pharmaceutical Manufacturers Association and 39 multinational pharmaceutical manufacturers brought a law suit against the Government of South Africa, alleging that the Medicines and Related Substances Control Amendment Act, No. 90 of 1997 (“Medicines Act“) violated TRIPS and the South African constitution. Provisions of the Medicines Act included generic substitution of off-patent medicines, transparent pricing for all medicines, and the parallel importation of patented medicines. Following extensive public outcry and the disclosure that the most contentious section of the Medicines Act was based on a draft legal text produced by the World International Property Organization (WIPO) Committee of Experts, the companies withdrew from the case in April 2001' [51].

#### Regulatory mechanisms to ensure access to quality-assured and affordable drugs

The offer by the Indian generic drug manufacturer Cipla to produce first-line triple FDC ART for less than a dollar a day in 2001 [53], the global mobilization based on a coordinated global HIV response, and the creation of the GFATM to provide ARVs to LMICs changed the approach to intellectual property rights for drugs [54]. The establishment of the WHO Prequalification Program in 2003 provided the necessary regulatory framework to provide quality-assured generic ARVs [55].

#### Doha Declaration on TRIPS and Public Health 2001

The Doha Declaration on TRIPS and Public Health at the World Trade Organization (WTO) ministerial meeting in 2001 signalled a shift in attitude toward intellectual property rights [55] and recognized their effect on drug prices. It affirmed the sovereign right of governments to take measures to protect public health, including but not limited to the use of compulsory licensing and parallel importation (Box 2).

**Box 2.** WTO Doha Declaration on Trade-Related Aspects of Intellectual Property Rights (TRIPS) and Public Health, 2001.

'We agree that the TRIPS Agreement does not and should not prevent Members from taking measures to protect public health. Accordingly, while reiterating our commitment to the TRIPS Agreement, we affirm that the Agreement can and should be interpreted and implemented in a manner supportive of WTO Members’ right to protect public health and, in particular, to promote access to medicines for all.
In this connection, we reaffirm the right of WTO Members to use, to the full, the provisions in the TRIPS Agreement, which provide flexibility for this purpose' [56].

The Doha Declaration led to the implementation of a special transition period for least developed country (LDC) members until 2033 for the implementation of pharmaceutical product patents and the protection of undisclosed test data. It waived the obligation to grant or enforce such rights, opening the way for the supply of generic ARVs, as many LDCs were already granting patent protection for medicines.

Countries were promised a solution to the restriction that TRIPS article 31(f) puts on the effective use of compulsory licensing by limiting the supply ‘predominantly for the domestic market’. This restriction caused problems for countries without sufficient production capacity that rely on importation of medicines to make effective use of compulsory licensing. In 2003 the TRIPS Council adopted the ‘August 30 decision’ setting out rules for compulsory licensing for export purposes, which formed the basis for an amendment to the TRIPS Agreement (article 31bis) adopted in 2005 and which went into force on 23 January 2017 [57].

#### From declaration to practice

One factor that contributed to the price decrease observed in ARVs has been the use by countries of the provisions of the Doha Declaration since 2001. Between 2001 and 2016, 89 countries had made use of TRIPS flexibilities in 176 cases. Of these, 100 cases concerned compulsory licenses, including for public non-commercial use [48]. These measures mainly involved the procurement of ARVs, and in some cases allowed for local production of ARVs. Pharmaceutical companies have also responded to these government measures by lowering their prices or agreeing to license their patents.

Between 2001 and 2016, 28 of the 34 WTO LDC members used paragraph 7 of the Doha Declaration to suspend the enforcement of pharmaceutical patents in procurement of ARVs [48]. This measure and the compulsory licenses offered important legal certainty for procurement agencies in supplying products that could be infringing a patent [58]. Therefore, these agencies supported the request from LDCs to further extend the deadline of the LDCs' transition period to implement their TRIPS obligations regarding pharmaceuticals beyond 2016. The Doha Declaration, however, holds additional options for LDCs and developing countries that have not been sufficiently explored (Box 3).

**Box 3.** Trade-Related Aspects of Intellectual Property Rights (TRIPS) Regional Waiver, 2003.

The ‘WTO August 30th Decision’ of 2003 created a regional waiver that allows exports under a compulsory license, without quantity restrictions, among countries that belong to a regional economic community (REC) of which at least half of the members are LDCs. Most RECs in Africa have a majority of LDC members. While LDCs of these RECs may continue to make use of the special LDC waiver, developing countries of RECs can issue compulsory licenses to locally produce or import generics and export or re-export, without quantity restrictions, within the REC, to harness economies of scale [59].

The political responses from the USA and the European Union to the use of TRIPS flexibilities by certain LMICs remain a significant obstacle to routine use of TRIPS flexibilities, especially for non-HIV drugs [60]. For example, when India issued a compulsory license in 2012 for a cancer medicine it provoked an out-of-cycle review by the US Trade Representative [61]. Similarly, recent attempts by Latin American countries to change existing laws to allow governments to issue compulsory licenses for NCD drugs have been strongly contested by pharmaceutical companies [62].

#### Turning the tide

In 2005, India amended its patent law to be compliant with the TRIPS Agreement. India then started to examine and grant pharmaceutical product patents that had been filed since 1995. India introduced strict patentability criteria, and its patent law has a provision enabling third parties to oppose the grant of a patent (‘pre-grant opposition’). This provision has launched procedures by generic manufacturers and civil society to limit the granting of undue patents and to protect the supply of low-cost newer ARVs, such as tenofovir disoproxil fumarate (TDF), as a first-line regimen.

However, the intellectual property (IP) landscape in India is rapidly changing, and more medicines developed since 1995 are being patented in India. To maintain generic production of new ARVs, licensing agreements and the use of TRIPS flexibilities will become more important. In 2010, the need for predictable and sustainable mechanism to ensure access to IP for generic manufacturers was recognized by UNITAID when it established the MPP. The MPP licences enable the manufacturing of generic ARVs and their sale in LMICs that cover 91% of adults living with HIV and up to 99% of paediatric formulations (Box 4).

**Box 4.** The first five years of the Medicines Patent Pool (MPP).

The MPP after five years of operation has signed voluntary licenses for 17 medicines, including one hepatitis C virus (HCV) drug with nine patent holders and 59 sub-licenses with 20 generic manufacturers. Generic companies with licenses from the MPP have supplied 14.6 million patient-years of WHO-recommended ARVs in 131 countries. The MPP licenses enable the manufacturing of generic adult formulations of ARVs and their sale in up to 131 countries where between 87% and 91% of PLHIV in LMIC live. Paediatric formulations cover up to 99% of children with HIV. The MPP agreements have saved US$391 million through lower prices of ARVs [63].

TRIPS flexibilities in the procurement of ARVs remain relevant for countries that cannot directly benefit from MPP licenses. The MPP licenses offer an indirect benefit to countries that are excluded from the license agreement by allowing licensees to export generic ARVs to such countries when they issue a compulsory license [64]. These insufficiently explored provisions in license agreements, combined with TRIPS flexibilities, offer a solution for affordable generic supply to all countries.

The use of patent grant oppositions, aimed at preventing blocking patents from being granted in the first place, is an approach that has been successfully applied for HIV and cancer across the world. In 2015 Médecins du Monde challenged Gilead’s patents related to the Hepatitis C medicine sofosbuvir (Sovaldi®) at the European Patent Office [65]. The Initiative for Medicines, Access & Knowledge, a non-profit organization focused on ensuring access to quality-assured and affordable medicines across the world, has filed petitions with the US Patent Trial and Appeal Board to challenge US patents related to sofosbuvir [66].

Other market exclusivities exist that have a similar effect to patents, and they prevent the marketing of a generic product for a certain period of time. Examples include data exclusivity that delays the registration of a generic product for a certain period, and market exclusivity. Such exclusivities may also hamper the effective use of TRIPS flexibilities [67].

## Conclusions

The price of APIs can be reduced by using quality-assured and affordable generic products to produce large volumes for multiple drugs. Market competition enables the price of APIs to be reduced, especially when future demand is quantified and based on good market forecasts. Improving the efficiency of the synthesis of APIs to improve the yield, identifying good-quality and cheaper raw materials, and optimizing the dose of the finished formulations are additional mechanims that can reduce the price of APIs and finished formulations. To achieve the 90-90-90, countries require access to quality-assured and affordable ARVs.

Non-HIV-related NCDs have become the predominant cause of death of older PLHIV in HICs [6]. Many PLHIV who start ART will eventually need access to NCD services and drugs. PLHIV are twice as likely to develop cardiovascular disease, and the population-attributable fraction for atherosclerotic cardiovascular disease is higher in LMICs than HICs [68]. NCDs are important causes of morbidity and mortality in their own right [69], and access to NCD health services and drugs is an integral part of providing universal health coverage [70]. People in LMICs need to have access to good-quality and affordable NCD drugs, and those factors that enable the production, distribution and access to quality-assured and affordable HIV drugs need to be reviewed and applied to NCD drugs [71].

TRIPS flexibilities are currently not being applied on the same scale for other diseases that lack the high profile and the level of political attention of HIV. The recent moves to sell common cancer drugs at a near-cost price in six African countries is a small step in the right direction [72], but it leaves countries dependent on what companies are willing to offer rather than what is needed in the country. Systematic approaches will be required to ensure access to new essential medicines that today are more widely patented. For instance, the high price of some of new cancer drugs is an issue in HICs [73]. The lessons from HIV show ways forward to a situation where licensing backed up with TRIPS safeguards become the norm.

Other avenues need to be explored to ensure that quality-assured and affordable drugs are available for people with communicable and non-communicable diseases irrespective of where they live [73,74]. This includes developing effective and efficient procurement and supply chain management systems [75]. In addition, it will require the development and implementation of legislative frameworks at country, regional and global levels that will support and ensure that these drugs are available on a sustainable basis for people in low, middle- and high-income countries. The question is, are policymakers and other stakeholders willing to develop the necessary legislative frameworks, implement them and implement the lessons learned to ensure access to quality-assured and affordable drugs for people who need them?

## Paper context

Increasing coverage of antiretroviral therapy in low- and middle-income countries will increase the life expectancy and number of people living with HIV. Many will develop non-communicable diseases. Generic drugs are cheaper compared with originator drugs. Licensing with TRIPS safeguards should become the norm to provide all drugs in low-, middle- and high-income countries within competitive generic markets to ensure sustainable availability of quality-assured and affordable drugs for communicable and non-communicable diseases.
